# Supplementary material for: An Exact Hypergraph Matching algorithm for posture identification in embryonic C. elegans
Source: PLoS One. 2022 Nov 29;17(11):e0277343. doi: 10.1371/journal.pone.0277343 (PMC9707761; doi:10.1371/journal.pone.0277343)
Supplement: S1 File — (PDF) [file pone.0277343.s001.pdf]

Supporting information

EHGM Pseudocode

EHGM requires as input the branching step $k \in \{1, 2, \ldots, n_1\}$, the dissimilarity tensors $\mathbf{Z}^{(d)}$, $d = 1, 2, \ldots, n_1$, the size k permutation set $\mathbf{P}$, and optionally an initial upper bound C_0 on the global minimum C^*. The dissimilarity tensors are calculated given the reference hypergraph of size n_1, either a previous frame hypergraph or a template hypergraph as described in section SI:*Model Fitting*. The n_2 input points and reference hypergraph are used to build the lower degree dissimilarity tensors $\mathbf{Z}^{(d)}, d \leq 2k$. The higher degree dissimilarity terms $d > 2k$ are calculated during the search as required. Algorithm 1 initializes the search from the first candidate set $\mathbf{Q}_1 = \mathbf{P}$. The search is easily parallelized via initializing several first branches at a time. Each explores a disjoint section of $\mathcal{X}$.

Algorithm 1 initializes arrays and variables to start the recursive branch search (Algorithm 2). Eligible branch candidates are subset from the general queue $\mathbf{P}$ into $\mathbf{Q}_m$ via the *Enqueue* procedure (Algorithm 3). Each $\mathbf{Q}_m$ contains the potential assignments for the next k terms that satisfy both the pruning constraints and assignment constraints specified by $\mathcal{X}$. The current assignment cost $\tilde{C}$ is checked against the current minimum C^* upon reaching a complete assignment. The Backtrack procedure (Algorithm 4) removes $\mathbf{k}_{m-1}$ from $\mathbf{Q}_{m-1}$ when the path from $\mathbf{k}_{m-1}$ is exhausted, which occurs when $\mathbf{Q}_m = \emptyset$. The recursion will continue until $\mathbf{Q}_1$ is empty, signaling the complete enumeration of the search space S_n.

Algorithm 1: EHGM

Input: $k, C_0, \mathbf{P}, \mathbf{Z}^{(1)}, \ldots, \mathbf{Z}^{(2k)}$
Output: $\mathbf{x}^*, C^* = f(\mathbf{x}^*)$
Initialization
$C^* \leftarrow C_0$
$\tilde{\mathbf{H}} = []$
$\tilde{\mathbf{I}} = []$
$\tilde{\mathbf{x}} \leftarrow \emptyset$
$\tilde{C} \leftarrow 0$
$m \leftarrow 1$
$\mathbf{Q}_1 \leftarrow Enqueue(\tilde{\mathbf{x}}, \mathbf{P}, \tilde{C}, C^*, 1)$
while $\mathbf{Q}_1 \neq \emptyset$ **do**
    $\mathbf{k}_1 \leftarrow \mathbf{Q}_1.pop()$
    $\hat{H}_1 \leftarrow H_1(\mathbf{k}_1 | \mathbf{Z}^{(1)}, \ldots, \mathbf{Z}^{(k)})$
    $\tilde{C} \leftarrow \hat{H}_1$
    $\tilde{\mathbf{H}}[1] = \hat{H}_1$
    $\tilde{\mathbf{x}} \leftarrow \tilde{\mathbf{x}} \cup \{\mathbf{k}_1\}$
    $m \mathrel{+}= 1$
    $Visit(\mathbf{P}, \tilde{\mathbf{x}}, \tilde{C}, C^*, m)$
end
Return: $\mathbf{x}^*, C^*$

Algorithm 2: Visit

Input: $\mathbf{P}, \tilde{\mathbf{x}}, \tilde{C}, C^*, m$
$\mathbf{Q}_m \leftarrow Enqueue(\tilde{\mathbf{x}}, \mathbf{P}, \tilde{C}, C^*, m)$
while $\mathbf{Q}_m \neq \emptyset$ **do**
  $\hat{\mathbf{k}}_m \leftarrow \mathbf{Q}_m.pop()$
  $\tilde{\mathbf{x}} \leftarrow \tilde{\mathbf{x}} \cup \{\hat{\mathbf{k}}_m\}$
  $m \mathrel{+}= 1$
  $\hat{H}_m \leftarrow H_m(\mathbf{k}_m | \tilde{\mathbf{x}}, \mathbf{Z}^{(1)}, \ldots, \mathbf{Z}^{(2k)})$
  $\tilde{\mathbf{H}}[m] = \hat{H}_m$
  $\tilde{C} \mathrel{+}= \hat{H}_m$
  if $m \geq 3$ **then**
    $\hat{I}_m \leftarrow I_m(\mathbf{k}_m | \tilde{\mathbf{x}}, \mathbf{Z}^{(2k+1)}, \ldots, \mathbf{Z}^{(mk)})$
    $\tilde{\mathbf{I}}[m] = \hat{I}_m$
    $\tilde{C} \mathrel{+}= \hat{I}_m$
  end
  if $m < M$ **then**
    $Visit(\mathbf{P}, \tilde{\mathbf{x}}, \tilde{C}, C^*, m)$
  else if $m = M$ **then**
    if $\tilde{C} \leq C^*$ **then**
      $\mathbf{x}^* \leftarrow \tilde{\mathbf{x}}$
      $C^* \leftarrow \tilde{C}$
    end
  $Backtrack(\tilde{\mathbf{x}}, \mathbf{Q}_{m-1}, m)$
end

Algorithm 3: Enqueue

Input: $\tilde{\mathbf{x}}, \mathbf{P}, \tilde{C}, C^*, m$
Output: $\mathbf{Q}_m$
$\mathbf{Q}_m \leftarrow \emptyset$
for $\mathbf{k} \in \mathbf{P}$ **do**
  if $(\mathbf{k} \cap \tilde{\mathbf{x}} = \emptyset) \wedge (\tilde{C} + H_m(\mathbf{k}|\tilde{\mathbf{x}}) < C^*)$ **then**
    $\mathbf{Q}_m \leftarrow \mathbf{Q}_m \cup \mathbf{k}$
  end
end

Algorithm 4: Backtrack

Input: $\tilde{\mathbf{x}}, \mathbf{Q}_{m-1}, m$
$\mathbf{Q}_{m-1} \leftarrow \mathbf{Q}_{m-1} \setminus \tilde{\mathbf{x}}_m$
$\tilde{\mathbf{x}} \leftarrow \{\hat{\mathbf{x}}_1, \hat{\mathbf{x}}_2, \ldots, \hat{\mathbf{x}}_{m-1}\}$
$\tilde{C} \leftarrow \Sigma_{j=1}^{m-1}(\mathbf{C}_m + \mathbf{I}_m)$

Hypergraphical Objective Decomposition

The hypergraphical optimization objective can be decomposed according to hyperedge multiplicity and branching step. The stratification enables efficient search via *EHGM*.

Theorem 1. *Assume an assignment problem objective f is in the form:*

$$
\begin{aligned}
f(X|\mathbf{Z}^{(1)}, \mathbf{Z}^{(2)}, \ldots, \mathbf{Z}^{(n_1)}) &= \sum_{l_1=1}^{n_1}\sum_{l_1'=1}^{n_2} \mathbf{Z}^{(1)}_{l_1 l_1'} x_{l_1 l_1'} + \sum_{l_1=1}^{n_1}\sum_{l_1'=1}^{n_2}\sum_{l_2=l_1+1}^{n_1}\sum_{l_2'=1}^{n_2} \mathbf{Z}^{(2)}_{l_1 l_1' l_2 l_2'} x_{l_1 l_1'} x_{l_2 l_2'} \\
&+ \sum_{l_1=1}^{n_1}\sum_{l_1'=1}^{n_2}\sum_{l_2=l_1+1}^{n_1}\sum_{l_2'=1}^{n_2}\sum_{l_3=l_2+1}^{n_1}\sum_{l_3'=1}^{n_2} \mathbf{Z}^{(3)}_{l_1 l_1' l_2 l_2' l_3 l_3'} x_{l_1 l_1'} x_{l_2 l_2'} x_{l_3 l_3'} + \ldots \\
&+ \sum_{l_1=1}^{n_1}\sum_{l_1'=1}^{n_2} \cdots \sum_{l_{n_1}=l_{n_1-1}+1}^{n_1}\sum_{l_{n_1}'=1}^{n_2} \mathbf{Z}^{(n_1)}_{l_1 l_1' \ldots l_{n_1} l_{n_1}'} x_{l_1 l_1'} \ldots x_{l_{n_1} l_{n_1}'}. \qquad (1)
\end{aligned}
$$

Then, for $k \in \{1, 2, \ldots, n_1\}$, the stratification fully describes the objective f after $M = \frac{n_1}{k}$ branches. Define of H_1, H_m, and I_m:

$$
H_1(\mathbf{K}_1|\mathbf{Z}^{(1)}, \mathbf{Z}^{(2)}, ..., \mathbf{Z}^{(k)}) :=
$$

$$
\sum_{i_1=1}^{k} \mathbf{Z}^{(1)}_{l_{i_1} l_{i_1}'} + \sum_{i_1=1}^{k}\sum_{i_2=i_1+1}^{k} \mathbf{Z}^{(2)}_{l_{i_1} l_{i_1}' l_{i_2} l_{i_2}'} + \ldots + \sum_{i_1=1}^{k}\sum_{i_2=i_1+1}^{k} \cdots \sum_{i_k=i_{k-1}+1}^{k} \mathbf{Z}^{(k)}_{l_1 l_{i_1}' l_{i_2} l_{i_2}' \ldots l_{i_k} l_{i_k}'},
$$

$$
H_m(\mathbf{K}_m|\mathbf{K}_1, ..., \mathbf{K}_{m-1}, \mathbf{Z}^{(1)}, ..., \mathbf{Z}^{(2k)}) :=
$$

$$
\sum_{i_1=(m-1)k+1}^{mk} \mathbf{Z}^{(1)}_{l_{i_1} l_{i_1}'} + \sum_{i_2=(m-1)k+1}^{mk}\sum_{i_1=1}^{i_2-1} \mathbf{Z}^{(2)}_{l_{i_1} l_{i_1}' l_{i_2} l_{i_2}'}
$$

$$
+ \sum_{i_3=(m-1)k+1}^{mk}\sum_{i_2=1}^{i_3-1}\sum_{i_1=1}^{i_2-1} \mathbf{Z}^{(3)}_{l_{i_1} l_{i_1}' l_{i_2} l_{i_2}' l_{i_3} l_{i_3}'} + \ldots + \sum_{i_{2k}=(m-1)k+1}^{mk}\sum_{i_{2k-1}=1}^{i_{2k}} \cdots \sum_{i_1=1}^{i_2-1} \mathbf{Z}^{(2k)}_{l_{i_1} l_{i_1}' \ldots l_{i_{2k}} l_{i_{2k}}'},
$$

and

$$I_m(\mathbf{K}_m|\mathbf{K}_1, \mathbf{K}_2, \dots, \mathbf{K}_{m-1}, \mathbf{Z}^{(2k+1)}, \dots, \mathbf{Z}^{(mk)}) := \sum_{d=2k+1}^{mk} \sum_{i_d=(m-1)k+1}^{mk} \sum_{i_{d-1}=1}^{i_d-1} \cdots \sum_{i_1=1}^{i_2-1} \mathbf{Z}^{(d)}_{l_{i_1} l'_{i_1} \dots l_{i_d} l'_{i_d}}.$$

Define $\Xi_m^{(d)}$*:*

$$\Xi_m^{(d)}(\mathbf{K}_m|\mathbf{K}_1, \mathbf{K}_2, \dots, \mathbf{K}_{m-1}, \mathbf{Z}^{(2k+1)}, \dots, \mathbf{Z}^{(mk)}) := \sum_{i_d=(m-1)k+1}^{mk} \sum_{i_{d-1}=1}^{i_d-1} \cdots \sum_{i_1=1}^{i_2-1} \mathbf{Z}^{(d)}_{l_{i_1} l'_{i_1} \dots l_{i_d} l'_{i_d}}$$

as each degree d *summand of* I_m*.*

Then, the degree n_1 *hypergraph matching objective* f *can be expressed*

$$f(X|\mathbf{Z}^{(1)}, \mathbf{Z}^{(2)}, \dots, \mathbf{Z}^{(n_1)}) = \sum_{m=1}^{m} H_m + \sum_{m=3}^{m} I_m.$$

Proof. First consider the single branching case $k = 1$. This yields $M = \frac{n_1}{k} = \frac{n_1}{1} = n_1$ branches. Each branch yields one assignment; i.e. $K_m = l'_m$ is assigned to vertex l_m. The initial branch selection rule H_1 can only utilize the first order term:

$$H_1(K_1|\mathbf{Z}^{(1)}) = \mathbf{Z}^{(1)}_{l_1 l'_1}.$$

Then the general selection rule for the second branch will: gather the first order costs for the second assignment as well as the quadratic (second order) costs between the first two assignments:

$$H_2(K_2|K_1, \mathbf{Z}^{(1)}, \mathbf{Z}^{(2)}) = \mathbf{Z}^{(1)}_{l_2 l'_2} + \sum_{i_2=2}^{2} \sum_{i_1=1}^{i_2} \mathbf{Z}^{(2)}_{l_{i_1} l'_{i_1} l_{i_2} l'_{i_2}} = \mathbf{Z}^{(1)}_{l_2} + \mathbf{Z}^{(2)}_{l_1 l'_1 l_2 l'_2}.$$

The third branching step will include H_3 and I_3. H_3 follows from H_2:

$$H_3(K_3|K_1, K_2, \mathbf{Z}^{(1)}, \mathbf{Z}^{(2)}) = \mathbf{Z}^{(1)}_{l_3 l'_3} + \sum_{i_2=3}^{3} \sum_{i_1=1}^{i_2-1} \mathbf{Z}^{(2)}_{l_{i_1} l'_{i_1} l_{i_2} l'_{i_2}} = \mathbf{Z}^{(1)}_{l_3 l'_3} + \mathbf{Z}^{(2)}_{l_1 l'_1 l_3 l'_3} + \mathbf{Z}^{(2)}_{l_2 l'_2 l_3 l'_3},$$

and

$$I_3(K_3|K_1, K_2, \mathbf{Z}^{(3)}) = \mathbf{Z}^{(3)}_{l_1 l'_1 l_2 l'_2 l_3 l'_3}.$$

Note that if $n_1 = 3$, then $H_1 + H_2 + H_3 + I_3$ fully describes the third order assignment problem:

$$\begin{aligned} H_1 + H_2 + H_3 + I_3 \\ &= \underbrace{\mathbf{Z}^{(1)}_{l_1 l'_1}}_{H_1} + \underbrace{\mathbf{Z}^{(1)}_{l_2 l'_2} + \mathbf{Z}^{(2)}_{l_1 l'_1 l_2 l'_2}}_{H_2} + \underbrace{\mathbf{Z}^{(1)}_{l_3 l'_3} + \mathbf{Z}^{(2)}_{l_1 l'_1 l_3 l'_3} + \mathbf{Z}^{(2)}_{l_2 l'_2 l_3 l'_3}}_{H_3} + \underbrace{\mathbf{Z}^{(3)}_{l_1 l'_1 l_2 l'_2 l_3 l'_3}}_{I_3} \\ &= \sum_{i_1=1}^{3} \mathbf{Z}^{(1)}_{l_{i_1} l'_{i_1}} + \sum_{i_1=1}^{3} \sum_{i_2=i_1+1}^{3} \mathbf{Z}^{(2)}_{l_{i_1} l'_{i_1} l_{i_2} l'_{i_2}} + \sum_{i_1=1}^{3} \sum_{i_2=i_1+1}^{3} \sum_{i_3=i_2+1}^{3} \mathbf{Z}^{(3)}_{l_{i_1} l'_{i_1} l_{i_2} l'_{i_2} l_{i_3} l'_{i_3}} \\ &= f(X|\mathbf{Z}^{(1)}, \mathbf{Z}^{(2)}, \mathbf{Z}^{(3)}). \quad (2) \end{aligned}$$

Now consider the extension to $n_1 = 4$, yielding a fourth degree assignment problem. The fourth branch will assign the next term, $K_4 = l'_4$. The terms H_4 and I_4 will then fully specify the fourth degree problem:

$$H_4(K_4|K_1, K_2, K_3, \mathbf{Z}^{(1)}, \mathbf{Z}^{(2)}) = \mathbf{Z}^{(1)}_{l_4 l'_4} + \mathbf{Z}^{(2)}_{l_1 l'_1 l_4 l'_4} + \mathbf{Z}^{(2)}_{l_2 l'_2 l_4 l'_4} + \mathbf{Z}^{(2)}_{l_3 l'_3 l_4 l'_4}.$$

The second aggregation rule I_4 will consider third order terms between branches $1, 2$ and 4 as well as the fourth order term using all four assignments:

$$I_4(K_4|K_1, K_2, K_3, \mathbf{Z}^{(3)}, \mathbf{Z}^{(4)}) = \mathbf{Z}^{(3)}_{l_1 l'_1 l_2 l'_2 l_4 l'_4} + \mathbf{Z}^{(3)}_{l_2 l'_2 l_3 l'_3 l_4 l'_4} + \mathbf{Z}^{(4)}_{l_1 l'_1 l_2 l'_2 l_3 l'_3 l_4 l'_4}.$$

The fourth branch terms can now be joined with the existing terms:

$$\begin{aligned}
&H_1 + H_2 + H_3 + I_3 + H_4 + I_4 = \\
&\quad \underbrace{\sum_{i_1=1}^{3} \mathbf{Z}^{(1)}_{l_{i_1} l'_{i_1}} + \sum_{i_1=1}^{3} \sum_{i_2=i_1+1}^{3} \mathbf{Z}^{(2)}_{l_{i_1} l'_{i_1} l_{i_2} l'_{i_2}} + \sum_{i_1=1}^{3} \sum_{i_2=i_1+1}^{3} \sum_{i_3=i_2+1}^{3} \mathbf{Z}^{(3)}_{l_{i_1} l'_{i_1} l_{i_2} l'_{i_2} l_{i_3} l'_{i_3}}}_{H_1+H_2+H_3+I_3} \\
&\quad + \underbrace{\mathbf{Z}^{(1)}_{l_4 l'_4} + \mathbf{Z}^{(2)}_{l_1 l'_1 l_4 l'_4} + \mathbf{Z}^{(2)}_{l_2 l'_2 l_4 l'_4} + \mathbf{Z}^{(2)}_{l_3 l'_3 l_4 l'_4}}_{H_4} + \underbrace{\mathbf{Z}^{(3)}_{l_1 l'_1 l_2 l'_2 l_4 l'_4} + \mathbf{Z}^{(3)}_{l_2 l'_2 l_3 l'_3 l_4 l'_4} + \mathbf{Z}^{(4)}_{l_1 l'_1 l_2 l'_2 l_3 l'_3 l_4 l'_4}}_{I_4} \\
&\quad = \sum_{i_1=1}^{4} \mathbf{Z}^{(1)}_{l_{i_1} l'_{i_1}} + \sum_{i_1=1}^{4} \sum_{i_2=i_1+1}^{4} \mathbf{Z}^{(2)}_{l_{i_1} l'_{i_1} l_{i_2} l'_{i_2}} + \sum_{i_1=1}^{4} \sum_{i_2=i_1+1}^{4} \sum_{i_3=i_2+1}^{4} \mathbf{Z}^{(3)}_{l_{i_1} l'_{i_1} l_{i_2} l'_{i_2} l_{i_3} l'_{i_3}} \\
&\qquad + \sum_{i_1=1}^{4} \sum_{i_2=i_1+1}^{4} \sum_{i_3=i_2+1}^{4} \sum_{i_4=i_3+1}^{4} \mathbf{Z}^{(4)}_{l_{i_1} l'_{i_1} l_{i_2} l'_{i_2} l_{i_3} l'_{i_3} l_{i_4} l'_{i_4}} \\
&\qquad = f(X|\mathbf{Z}^{(1)}, \mathbf{Z}^{(2)}, \mathbf{Z}^{(3)}, \mathbf{Z}^{(4)}). \qquad (3)
\end{aligned}$$

Now consider the arbitrary *m+1*st branch. This addition will yield the full objective for an assignment problem of size $m + 1$ up to degree $m + 1$:

$$\begin{aligned}
&\sum_{p=1}^{m+1} H_p + \sum_{p=3}^{m+1} I_p = \sum_{p=1}^{m} H_p + \sum_{p=3}^{m} I_p + H_{m+1} + I_{m+1} = \\
&\underbrace{\sum_{i_1=1}^{m} \mathbf{Z}^{(1)}_{l_{i_1} l'_{i_1}} + \sum_{i_1=1}^{m} \sum_{i_2=i_1+1}^{m} \mathbf{Z}^{(2)}_{l_{i_1} l'_{i_1} l_{i_2} l'_{i_2}} + \cdots + \sum_{i_1=1}^{m} \sum_{i_2=i_1+1}^{m} \cdots \sum_{i_m=i_{m-1}+1}^{m} \mathbf{Z}^{(m)}_{l_{i_1} l'_{i_1} l_{i_2} l'_{i_2} \ldots l_{i_m} l'_{i_m}}}_{\sum_{p=1}^{m} H_p + \sum_{p=3}^{m} I_p} \\
&\qquad + \underbrace{\mathbf{Z}^{(1)}_{l_{i_{m+1}} l'_{i_{m+1}}} + \sum_{i_1=1}^{m} \mathbf{Z}^{(2)}_{l_{i_1} l'_{i_1} l_{m+1} l'_{m+1}}}_{H_{m+1}} + \underbrace{\sum_{d=3}^{m+1} \Xi^{(d)}_{m+1}}_{I_{m+1}}. \qquad (4)
\end{aligned}$$

It is sufficient to show each degree $d \in \{1, 2, \ldots, m + 1\}$ hyperedge is fully accounted for across all $m + 1$ points to prove the *m+1*st branch satisfies the objective *f*. The hyperedge costs across all points will be decomposed into three disjoint sets, and each set considered at a time:

$$\{1\}, \{2\}, \{3, \ldots, m\}, \{m + 1\}.$$

The first and final of the four cases are trivial. The first degree terms are enumerated via the first term in H_{m+1}, while $Xi_{m+1}^{(m+1)}$ explicitly addresses the degree $m+1$ hyperedge comprising all assignments: $\mathbf{Z}^{(m+1)}_{l_1 l'_1 l_2 l'_2 \ldots l_{m+1} l'_{m+1}}$. We will focus on the second and third cases. The degree $d = 2$ terms are formed by the addition of branch $m+1$ are considered in term H_{m+1}:

$$\sum_{i_1=1}^{m} \sum_{i_2=i_1+1}^{m} \mathbf{Z}^{(2)}_{l_{i_1} l'_{i_1} l_{i_2} l'_{i_2}} + \sum_{i_1=1}^{m} \mathbf{Z}^{(2)}_{l_{i_1} l'_{i_1} l_{i_{m+1}} l'_{i_{m+1}}} = \sum_{i_1=1}^{m+1} \sum_{i_2=i_1+1}^{m+1} \mathbf{Z}^{(2)}_{l_{i_1} l'_{i_1} l_{i_2} l'_{i_2}}.$$

Let $d \in \{3, \ldots, m\}$. The completion is similar to the $d = 2$ degree case; however, the term $\Xi^{(d)}_{m+1}$ in I_{m+1} address higher degree hyperedges up to and including degree m concerning branch $m+1$:

$$\sum_{i_1=1}^{m} \cdots \sum_{i_d=i_{d-1}+1}^{m} \mathbf{Z}^{(d)}_{l_{i_1} l'_{i_1} \ldots l_{i_d} l'_{i_d}} + \Xi^{(d)}_{m+1} = \sum_{i_1=1}^{m+1} \cdots \sum_{i_d=i_{d-1}+1}^{m+1} \mathbf{Z}^{(d)}_{l_{i_1} l'_{i_1} \ldots l_{i_d} l'_{i_d}}.$$

Therefore, the $(m+1)^{st}$ step fully accrues the objective f:

$$\sum_{p=1}^{m+1} H_p + \sum_{p=3}^{m+1} I_p = f(X|\mathbf{Z}^{(1)}, \mathbf{Z}^{(2)}, \ldots, \mathbf{Z}^{(m+1)}).$$

Then inductively, the stratification holds such that:

$$\sum_{m=1}^{n_1} H_m + \sum_{m=3}^{n_1} I_m = f(X|\mathbf{Z}^{(1)}, \mathbf{Z}^{(2)}, \ldots \mathbf{Z}^{(n_1)}).$$

Now consider the plural branching rule $k > 1$. The proof will follow from the single assignment branching case. The base case at the fourth branch will be established, followed by the induction hypothesis demonstrating the branching from m to $m+1$. First, define the terms H_1, H_2, H_3, I_3, H_4, and I_4:

$$H_1 = \sum_{i_1=1}^{k} \mathbf{Z}^{(1)}_{l_{i_1} l'_{i_1}} + \sum_{i_1=1}^{k} \sum_{i_2=i_1+1}^{k} \mathbf{Z}^{(2)}_{l_{i_1} l'_{i_1} l_{i_2} l'_{i_2}} + \ldots + \sum_{i_1=1}^{k} \sum_{i_2=i_1+1}^{k} \cdots \sum_{i_k=i_{k-1}+1}^{k} \mathbf{Z}^{(k)}_{l_{i_1} l'_{i_1} l_{i_2} l'_{i_2} \ldots l_{i_k} l'_{i_k}}, \tag{5}$$

$$H_2 = \sum_{i_1=k+1}^{2k} \mathbf{Z}^{(1)}_{l_{i_1} l'_{i_1}} + \sum_{i_2=k+1}^{2k} \sum_{i_1=1}^{i_2-1} \mathbf{Z}^{(2)}_{l_{i_1} l'_{i_1} l_{i_2} l'_{i_2}} + \ldots + \sum_{i_{2k}=k+1}^{2k} \cdots \sum_{i_2=1}^{i_3-1} \sum_{i_1=1}^{i_2-1} \mathbf{Z}^{(2k)}_{l_{i_1} l'_{i_1} \ldots l_{i_{2k}} l'_{i_{2k}}}, \tag{6}$$

$$H_3 = \sum_{i_1=2k+1}^{3k} \mathbf{Z}^{(1)}_{l_{i_1} l'_{i_1}} + \sum_{i_2=2k+1}^{3k} \sum_{i_1=1}^{i_2-1} \mathbf{Z}^{(2)}_{l_{i_1} l'_{i_1} l_{i_2} l'_{i_2}} + \ldots + \sum_{i_{2k}=2k+1}^{3k} \cdots \sum_{i_1=1}^{i_2-1} \mathbf{Z}^{(2k)}_{l_{i_1} l'_{i_1} \ldots l_{i_{2k}} l'_{i_{2k}}}, \tag{7}$$

$$I_3 = \sum_{d=2k+1}^{3k} \Xi^{(d)}_3, \tag{8}$$

$$H_4 = \sum_{i_1=3k+1}^{4k} \mathbf{Z}^{(1)}_{l_{i_1} l'_{i_1}} + \sum_{i_2=3k+1}^{4k} \sum_{i_1=1}^{i_2-1} \mathbf{Z}^{(2)}_{l_{i_1} l'_{i_1} l_{i_2} l'_{i_2}} + \ldots + \sum_{i_{2k}=3k+1}^{4k} \cdots \sum_{i_1=1}^{i_2-1} \mathbf{Z}^{(2k)}_{l_{i_1} l'_{i_1} \ldots l_{i_{2k}} l'_{i_{2k}}}, \tag{9}$$

and

$$I_4 = \sum_{d=3k+1}^{4k} \Xi_4^{(d)}. \tag{10}$$

The terms presented thus far for the general $k > 1$ case fully describe all terms concerning assignments $1, 2, \ldots 4k$ up to degree $4k$. The hyperedge multiplicities will again be partitioned into disjoint groups:

$$\{1\}, \{2, \ldots, k\}, \{k+1, \ldots, 2k\}, \{2k+1, \ldots, 3k\}, \{3k+1, \ldots, 4k\}.$$

The first case is trivial, just as in the single assignment branching ($k = 1$) proof. Unary terms are accounted for in the first summand of each H_m. Then, consider $d \in \{2, \ldots, k\}$:

$$\underbrace{\sum_{i_1=1}^{k} \cdots \sum_{i_d=i_{d-1}+1}^{k} \mathbf{Z}^{(d)}_{l_{i_1} l'_{i_1} \ldots l_{i_d} l'_{i_d}}}_{H_1} + \underbrace{\sum_{i_d=k+1}^{2k} \sum_{i_{d-1}=1}^{i_d-1} \cdots \sum_{i_1=1}^{i_2-1} \mathbf{Z}^{(d)}_{l_{i_1} l'_{i_1} \ldots l_{i_d} l'_{i_d}}}_{H_2}$$
$$+ \underbrace{\sum_{i_d=2k+1}^{3k} \sum_{i_{d-1}=1}^{i_d-1} \cdots \sum_{i_1=1}^{i_2-1} \mathbf{Z}^{(d)}_{l_{i_1} l'_{i_1} \ldots l_{i_d} l'_{i_d}}}_{H_3} + \underbrace{\sum_{i_d=3k+1}^{4k} \sum_{i_{d-1}=1}^{i_d-1} \cdots \sum_{i_1=1}^{i_2-1} \mathbf{Z}^{(d)}_{l_{i_1} l'_{i_1} \ldots l_{i_d} l'_{i_d}}}_{H_4}$$
$$= \sum_{i_1=1}^{4k} \sum_{i_2=i_1+1}^{4k} \cdots \sum_{i_d=i_{d-1}+1}^{4k} \mathbf{Z}^{(d)}_{l_{i_1} l'_{i_1} \ldots l_{i_d} l'_{i_d}}.$$

The proof for degree $d \in \{k+1, \ldots, 2k\}$ follows immediately from the grouping presented above, but without the initial branch selection rule term H_1. Next, assume $d \in \{2k+1, \ldots, (m-1)k\}$. Degree d hyperedge dissimilarities will be contained in both I_3 and I_4 terms:

$$\underbrace{\sum_{i_d=2k+1}^{3k} \sum_{i_{d-1}=1}^{i_d-1} \cdots \sum_{i_1=1}^{i_2-1} \mathbf{Z}^{(d)}_{l_{i_1} l'_{i_1} \ldots l_{i_d} l'_{i_d}}}_{I_3} + \underbrace{\sum_{i_d=3k+1}^{4k} \sum_{i_{d-1}=1}^{i_d-1} \cdots \sum_{i_1=1}^{i_2-1} \mathbf{Z}^{(d)}_{l_{i_1} l'_{i_1} \ldots l_{i_d} l'_{i_d}}}_{I_4}$$
$$= \sum_{i_1=1}^{4k} \cdots \sum_{i_{d-1}=i_{d-2}+1}^{4k} \sum_{i_d=i_{d-1}+1}^{4k} \mathbf{Z}^{(d)}_{l_{i_1} l'_{i_1} \ldots l_{i_d} l'_{i_d}}.$$

Since $d \leq 2k+1$, the terms only appear in the third branch term I_3 when the assignment $2k+1$ is committed. The final set arises from the definition of I_4 which accrues hyperedges of degree $d \in \{3k+1, \ldots, 4k\}$ across assignments in branches $m = 1, 2, 3, 4$. The base case is fully established for the arbitrary $k > 1$ case. The final step of the proof is to establish the extension of the m+1st branch:

$$\sum_{p=1}^{m+1} H_p + \sum_{p=3}^{m+1} I_p = \sum_{p=1}^{m} H_p + \sum_{p=3}^{m} I_p + H_{m+1} + I_{m+1} =$$

$$\underbrace{\sum_{i_1=1}^{mk} \mathbf{Z}^{(1)}_{l_{i_1} l'_{i_1}} + \sum_{i_1=1}^{mk} \sum_{i_2=i_1+1}^{mk} \mathbf{Z}^{(2)}_{l_{i_1} l'_{i_1} l_{i_2} l'_{i_2}} + \cdots + \sum_{i_1=1}^{mk} \sum_{i_2=i_1+1}^{mk} \cdots \sum_{i_{mk}=i_{mk-1}+1}^{mk} \mathbf{Z}^{(mk)}_{l_{i_1} l'_{i_1} \ldots l_{i_{mk}} l'_{i_{mk}}}}_{\sum_{p=1}^{m} H_p + \sum_{p=3}^{m} I_p}$$

$$+ \underbrace{\sum_{i_1=mk+1}^{(m+1)k} \mathbf{Z}^{(1)}_{l_{i_1} l'_{i_1}} + \ldots + \sum_{i_{2k}=mk+1}^{(m+1)k} \sum_{i_{2k-1}=1}^{i_{2k}-1} \cdots \sum_{i_1=1}^{i_2-1} \mathbf{Z}^{(2k)}_{l_{i_1} l'_{i_1} \ldots l_{i_{2k}} l'_{i_{2k}}}}_{H_{m+1}} + \underbrace{\sum_{d=2k+1}^{(m+1)k} \Xi^{(d)}_{m+1}}_{I_{m+1}} . \quad (11)$$

The $(m+1)k$ hyperedge multiplicities will be stratified into four groups:

$$\{1\}, \{2, \ldots, 2k\}, \{2k+1, \ldots, mk\}, \{mk+1, \ldots, (m+1)k\}.$$

Just as in the singular $k = 1$ case, the proof for the first and last groups are trivial. The unary terms are again evident from the first term in H_{m+1}, while the $mk + 1 \leq d \leq (m+1)k$ terms in I_{m+1} fully encapsulates the fourth group. The steps in the remaining two cases will follow that of the $k = 1$ case.

First, assume $d \in \{2, \ldots, 2k\}$. The extension of the *m+1*st branch uses exclusively the selection rule H_{m+1}:

$$\underbrace{\sum_{i_1=1}^{mk} \cdots \sum_{i_{d-1}=i_{d-2}+1}^{mk} \sum_{i_d=i_{d-1}+1}^{mk} \mathbf{Z}^{(d)}_{l_{i_1} l'_{i_1} \ldots l_{i_d} l'_{i_d}}}_{\sum_{p=1}^{m} H_m} + \underbrace{\sum_{i_d=mk+1}^{(m+1)k} \sum_{i_{d-1}=1}^{i_d-1} \cdots \sum_{i_1=1}^{i_2-1} \mathbf{Z}^{(d)}_{l_{i_1} l'_{i_1} \ldots l_{i_d} l'_{i_d}}}_{H_{m+1}} =$$

$$\sum_{i_1=1}^{(m+1)k} \cdots \sum_{i_{d-1}=i_{d-2}+1}^{(m+1)k} \sum_{i_d=i_{d-1}+1}^{(m+1)k} \mathbf{Z}^{(d)}_{l_{i_1} l'_{i_1} \ldots l_{i_d} l'_{i_d}} . \quad (12)$$

Next, assume $d \in \{2k+1, \ldots, mk\}$. These terms are captured in I_{m+1} using each definition of $\Xi^{(d)}_{m+1}$:

$$\sum_{i_1=1}^{mk} \cdots \sum_{i_{d-1}=i_{d-2}+1}^{mk} \sum_{i_d=i_{d-1}+1}^{mk} \mathbf{Z}^{(d)}_{l_{i_1} l'_{i_1} \ldots l_{i_d} l'_{i_d}} + \underbrace{\sum_{i_d=mk+1}^{(m+1)k} \sum_{i_{d-1}=1}^{i_d-1} \cdots \sum_{i_1=1}^{i_2-1} \mathbf{Z}^{(d)}_{l_{i_1} l'_{i_1} \ldots l_{i_d} l'_{i_d}}}_{I_{m+1}} =$$

$$\sum_{i_1=1}^{(m+1)k} \cdots \sum_{i_{d-1}=i_{d-2}+1}^{(m+1)k} \sum_{i_d=i_{d-1}+1}^{(m+1)k} \mathbf{Z}^{(d)}_{l_{i_1} l'_{i_1} \ldots l_{i_d} l'_{i_d}} . \quad (13)$$

All four results together show that every degree hyperedge $1, \ldots, (m+1)k$ is accounted for in the *m+1*st branch, thus proving the induction hypothesis:

$$\sum_{p=1}^{m+1} H_p + \sum_{p=3}^{m+1} I_p = f(X|\mathbf{Z}^{(1)}, \mathbf{Z}^{(2)}, \ldots \mathbf{Z}^{((m+1)k)}).$$

The Mth branch completes the degree n_1 assignment problem of size n_1. For any $k \in \{1, 2, \dots n_1\}$, the selection and aggregation rules yield the full degree n_1 assignment problem objective:

$$\sum_{p=1}^{M} H_p + \sum_{p=3}^{M} I_p = f(X|\mathbf{Z}^{(1)}, \mathbf{Z}^{(2)}, \dots \mathbf{Z}^{(n_1)}).$$

□

Convergence & Exactness of *EHGM*

Theorem 2. *EHGM (algorithm 1) will converge to a globally optimal solution of the following hypergraph matching optimization problem given input $k \in \{1, 2, \dots, n_1\}$:*

$$\begin{aligned}\min_{X \in \mathcal{X}} \sum_{l_1=1}^{n_1} \sum_{l'_1=1}^{n_2} \mathbf{Z}^{(1)}_{l_1 l'_1} x_{l_1 l'_1} + \sum_{l_1=1}^{n_1} \sum_{l'_1=1}^{n_2} \sum_{l_2=l_1+1}^{n_1} \sum_{l'_2=1}^{n_2} \mathbf{Z}^{(2)}_{l_1 l'_1 l_2 l'_2} x_{l_1 l'_1} x_{l_2 l'_2} \\ + \sum_{l_1=1}^{n_1} \sum_{l'_1=1}^{n_2} \sum_{l_2=l_1+1}^{n_1} \sum_{l'_2=1}^{n_2} \sum_{l_3=l_2+1}^{n_1} \sum_{l'_3=1}^{n_2} \mathbf{Z}^{(3)}_{l_1 l'_1 l_2 l'_2 l_3 l'_3} x_{l_1 l'_1} x_{l_2 l'_2} x_{l_3 l'_3} + \dots \\ + \sum_{l_1=1}^{n_1} \sum_{l'_1=1}^{n_2} \dots \sum_{l_{n_1}=l_{n_1-1}+1}^{n_1} \sum_{l'_{n_1}=1}^{n_2} \mathbf{Z}^{(n_1)}_{l_1 l'_1 \dots l_{n_1} l'_{n_1}} x_{l_1 l'_1} \dots x_{l_{n_1} l'_{n_1}} \end{aligned} \quad (14)$$

where $\mathcal{X}$ is defined:

$$\mathcal{X} = \{X \in \{0,1\}^{n_1 \times n_2} : \forall j, \sum_{i=1}^{n_1} x_{l_i l'_j} = 1, \forall i \sum_{j=1}^{n_2} x_{l_i l'_j} \leq 1\}.$$

Proof. First, we will show *EHGM* converges, then it will be proven that the converged solution is globally optimal.

The search begins with initializing queue $\mathbf{Q}_1 = \mathbf{P}$. The algorithm terminates with the exhaustion of $\mathbf{Q}_1$. Each set $\mathbf{Q}_m \subset \mathbf{P}$ contains feasible k-assignments conditioned on the assignment constraints and costs $\tilde{C}, C^*$. *Backtrack* (Algorithm 4) removes $\mathbf{x}_m$ from $\mathbf{Q}_{m-1}$ upon enumeration of $\mathbf{Q}_m$. The recursion then falls back to selecting from branch $m-1$, eventually exhausting $\mathbf{Q}_{m-1}$ just as in the enumeration of $\mathbf{Q}_m$. This recursion continues until the first branch $\mathbf{k}_1 \in \mathbf{Q}_1$ is removed, signaling the exploration of all assignments originating with the k-tuple $\mathbf{k}_1$. The exploration is repeated for each $\mathbf{k}_1 \in \mathbf{Q}_1$. Thus, all possible assignments $X \in \mathcal{X}$ are explored via the branching scheme.

Assignments accrue a monotonically increasing cost $\tilde{C}$ to be compared to C^* with accompanying assignment $\mathbf{x}^*$ at each branch. A complete assignment then drops the last k assignments from $\tilde{\mathbf{x}}$, initializing the backwards recursion, emptying $\mathbf{Q}_m$ until $\mathbf{k}^{(n_k)}_{m-1} \in \mathbf{Q}_{m-1}$ is exhausted. There are at most $|\mathbf{Q}_m| \leq n_k$ viable permutations at branch m. Each possible branch is evaluated from $\tilde{\mathbf{x}} = [\mathbf{k}_1, \mathbf{k}_2, \dots \mathbf{k}_{m-1}]$. The $(m-1)^{st}$ branch $\mathbf{k}_{m-1} \in \mathbf{Q}_{m-1}$ is removed from $\mathbf{Q}_{m-1}$ upon exhaustion of $\mathbf{Q}_m$:

$$\begin{aligned}\tilde{\mathbf{x}}^{(m)}_1 &= [\mathbf{k}^{(1)}_1, \mathbf{k}^{(1)}_2, \dots \mathbf{k}^{(1)}_{m-1}, \mathbf{k}^{(1)}_m] \\ \tilde{\mathbf{x}}^{(m)}_2 &= [\mathbf{k}^{(1)}_1, \mathbf{k}^{(1)}_2, \dots \mathbf{k}^{(1)}_{m-1}, \mathbf{k}^{(2)}_m] \\ &\dots \\ \tilde{\mathbf{x}}^{(m)}_{n_k} &= [\mathbf{k}^{(1)}_1, \mathbf{k}^{(1)}_2, \dots \mathbf{k}^{(1)}_{m-1}, \mathbf{k}^{(n_k)}_m].\end{aligned}$$

Each of the n_k possible final branches from $\mathbf{k}_{m-1}^{(1)}$ is explored, then $\mathbf{k}_{m-1}^{(1)}$ is removed from $\mathbf{Q}_{m-1}$.

The process follows for the Mth branch, exhausting viable assignment sets until $\mathbf{k}_{M-1}$ is removed. The recursion follows inductively back to the exhaustion of $\mathbf{Q}_1$, signaling the end of the search. Thus, all possible assignments $X \in \mathcal{X}$ are explored via the branching scheme.

The convergent and exhaustive algorithm will yield a globally optimal solution $C^* = f(\mathbf{x}^*)$ after exhausting $\mathbf{Q}_1$. As proven above the additive decomposition of the cost structure (equation 1) is proven to be satisfied by summing all selection and aggregation rule terms. Assume an uninformed initialization $C^* = \infty$. Then the first pass will greedily take the best permutation from the first set $\mathbf{Q}_1$: $\mathbf{k}_1^{(1)}$, and the best from the second set given it does not conflict with $\mathbf{k}_1^{(1)}$: $\mathbf{k}_2^{(2|1)}$. This process will continue until the first complete assignment:
$\tilde{\mathbf{x}} = [\mathbf{k}_1^{(1)}, \mathbf{k}_2^{(2|1)}, \mathbf{k}_3^{(3|2,1)}, \dots, \mathbf{k}_M^{(M|(M-1),\dots,1)}]$ with $\tilde{C} = f(\tilde{\mathbf{x}})$. The first *Backtrack* removes $\mathbf{k}_M^{(M|(M-1),\dots,1)}$, and the Mth *Visit* call will exhaust $\mathbf{Q}_M$. Subsequent *Enqueue* calls will limit only allow branches that satisfy both the assignment constraints and the updated selection rule cost (Algorithm 3). This follows that any k-tuple of assignments $\mathbf{k}_m^{(j)}$ such that for $\tilde{\mathbf{x}} = [\mathbf{k}_1^{(j_1)}, \mathbf{k}_2^{(j_2|j_1)}, \dots, \mathbf{k}_m^{(j_d|j_{(m-1)},\dots j_1)}]$:

$$\tilde{C} + H_m(\tilde{\mathbf{x}}_{m-1}, \mathbf{k}_m^{(j_d|j_{(m-1)},\dots j_1)}) < C^*.$$

The additive decomposition of the objective paired with the assumed non-negativity of the dissimilarity tensors $\mathbf{Z}^{(j)}$ results in each branch monotonically increasing $\tilde{C}$:

$$\tilde{C} + H_m(\tilde{\mathbf{x}}_{m-1}, \mathbf{k}_m^{(j_d|j_{(m-1)},\dots j_1)}) + I_m(\mathbf{k}_1^{(j_1)}, \mathbf{k}_2^{(j_2|j_1)}, \dots, \mathbf{k}_m^{(j_d|j_{(m-1)},\dots j_1)}) \geq \tilde{C}.$$

The convergent search will thus eliminate all paths that are not globally optimal. Incrementally updating the reserved solution $\mathbf{x}^*$ with cost C^* expedites convergence as each replacement is necessarily a better solution. The resulting $\mathbf{x}^*$ and corresponding cost C^* are such that at no other full assignment $\tilde{\mathbf{x}}$ can replace $\mathbf{x}^*$, by definition a globally optimal solution of *f*.

□

Model Fitting

Expert annotations are used to derive features such that the correct assignment consistently achieves a minimal cost across the training set. Features can be engineered and analyzed in context of point set matching just as in traditional supervised learning tasks.

Features are expressed as attributes over hyperedge multiplicities $d = 1, 2, \dots, n$. Hyperedge features $g_s^{(d)}$, $s = 1, \dots, n_d$ are given as input. Each feature $g_s^{(d)}$ assumes a Gaussian distribution, and if $n_d \geq 2$ the features are modeled as a multivariate Gaussian distribution. Measurements from the data are used to derive estimates of the parameters of the Gaussian distributions. The most common application in heuristic approaches is to use the previous frame's feature values as the centers of the distributions. This standard approach is effective for simple lower variance features. However, higher degree features may have more variation frame-to-frame. Mean estimates across the training data can better account for macroscopic patterns in more complex features. The variances are then estimated from the feature values across the training set. The dissimilarity costs arise from functions attributed to hyperedges quantified by parametric assumptions.

The dissimilarity tensors $\mathbf{Z}^{(d)}$ can now be expressed as a function of the n_d features of hyperedge d. A partial assignment up to degree d: $[(l_1, \ldots, l_d) \mapsto (l'_1, \ldots, l'_d)]$ invokes a cost according to the n_d features: $\sum_{s=1}^{n_d} g_s^{(d)}$. The expected values: $\sum_{s=1}^{n_d} \bar{g}_s^{(d)}$ are calculated in aggregate from training data for higher variance patterns:

$$\bar{g}_s^{(d)} = \frac{\sum_{L=1}^{N} g_s^{(d)}(X_L, \mathbf{X}_L)}{N}. \quad (15)$$

where X_L and $\mathbf{X}_L$ are the correct permutation and observed point set, respectively, for sample L. The variance-covariance matrix uses estimated means to estimate variances and covariances among feature measurements in the annotated data:

$$\hat{\sigma}_{a,b}^{(d)} = \sum_{L=1}^{N} (g_a^{(d)}(X_L, \mathbf{X}_L) - \bar{g}_a^{(d)})(g_b^{(d)}(X_L, \mathbf{X}_L) - \bar{g}_b^{(d)}), \quad (16)$$

$$\hat{\mathbf{\Sigma}}_g^{(d)} = \left\| \begin{bmatrix} \hat{\sigma}_{1,1}^{(d)} & \hat{\sigma}_{1,2}^{(d)} & \ldots & \hat{\sigma}_{1,n_d}^{(d)} \\ \hat{\sigma}_{2,1}^{(d)} & \hat{\sigma}_{2,2}^{(d)} & \ldots & \hat{\sigma}_{2,n_d}^{(d)} \\ \ldots & \ldots & \ldots & \ldots \\ \hat{\sigma}_{n_d,1}^{(d)} & \hat{\sigma}_{n_d,2}^{(d)} & \ldots & \hat{\sigma}_{n_d,n_d}^{(d)} \end{bmatrix} \right\|.$$

The selection rule tensor dissimilarity tensors $\mathbf{Z}^{(d)} \in R^{\overbrace{n \times n, \ldots, \times n}^{2d}}$ use both sets of estimates to compute costs. The Mahalanobis distance is used to describe the scaled distance between the observed attributed hyperedge to an estimated feature description. Let $\mathbf{g}^{(d)} = [g_1^{(d)}, g_2^{(d)}, \ldots, g_{n_d}^{(d)}]'$ and $\bar{\mathbf{g}}^{(d)} = [\bar{g}_1^{(d)}, \bar{g}_2^{(d)}, \ldots, \bar{g}_{n_d}^{(d)}]'$. Then $\mathbf{Z}^{(d)}$ evaluated at the assignment $[(l_1, \ldots, l_d) \mapsto (l'_1, \ldots, l'_d)]$ can be expressed:

$$\mathbf{Z}_{l_1 l'_1 l_2 l'_2 \ldots l_d l'_d}^{(d)} = (\mathbf{g}^{(d)} - \bar{\mathbf{g}}^{(d)})'(\hat{\mathbf{\Sigma}}_g^{(d)})^{-1}(\mathbf{g}^{(d)} - \bar{\mathbf{g}}^{(d)}). \quad (17)$$

Posture Modeling

This section aims to introduce feature visualizations and discuss the effectiveness of various features for posture assignment.

Consider an image captured at time t with $n = 20$ located nuclei centroids. The coordinates can be stored as $\mathbf{X} \in R^{n,3}$ which $\mathbf{X}_i = [x_i, y_i, z_i]$ representing the ith centroid in R^3. Then let $1, 3, 5, \ldots 19$ be in the indices of the left side, and $2, 4, 6, \ldots 20$ be the indices of the right side. Then the cell nuclei are paired $(1, 2)$ for the tail pair, then $(2, 3), (4, 5), (6, 7), \ldots (19, 20)$ for the body pairs. Let $\mathbf{L} = (\mathbf{l}_1, \mathbf{l}_2, \ldots \mathbf{l}_m)$ denote the left side locations, and similarly $\mathbf{R}$ the right.

The sampled worm embryos tend to develop at similar rates. However, the occurrence of the first twitch, a point in development that triggers rapid physical changes, varies embryo to embryo. Thus a time normalization is applied in effort to compare data from the different samples. Each worm's time to first twitch is measured s_w, as well as hatch time h_w for worm $w = 1, 2, \ldots 10$. The time points for each sample are indexed $k = 1, 2, \ldots n_w$. Now, each volume's imaging time t_{wk} is normalized to the $[0, 1]$ scale via the following formula:

$$z_{wk} = \frac{t_{wk} - s_w}{h_w - s_w}.$$

Each time point $z_{wk} \in [0, 1]$ is scaled such that $z_{wk} = 0$ represents first twitch, and $z_{wk} = 1$ hatching. All plots will feature the normalized time of observation z_{wk} on the horizontal axis. The y axis unit will vary plot to plot. Distances will be measured in microns, μm, angles measured in degrees, $\theta \in [0, 360]$. Plots with multiple subplots will

measure pairwise features, features defined by linking a length two sequence of pairs, or features defined by a length three sequence of pairs. The leftmost plot will depict features for the tail or sequence starting from the tail, while the rightmost will depict the feature ending with the head pair, at the anterior of the worm. Features are calculated conditioned on stage of development in binned intervals of length .05 as well as the region in the worm measured by pair index.

Sides

The quadratic model uses scaled distances between pairs of nuclei. The first feature to analyze is the distance between paired cell nuclei (S1 Fig 1-A):

$$\mathbf{PD}_i = \|\mathbf{L}_i - \mathbf{R}_i\|_2. \tag{18}$$

This can be biologically interpreted as the width of the worm measured at the sampled nuclei locations. As mentioned above, the first subplot illustrates distances in microns between nuclei centroids of the tail pair for each observation. The distance between nuclei in the tail pair (left-most panel) is used for the initial pair selection rule H_1 across all models. The second set of distances form along the left and right sides of the worm (S1 Fig 1-B). The lengths of chords between successive nuclei on each side are calculated: $\|\mathbf{L}_{i+1} - \mathbf{L}_i\|_2$ and $\|\mathbf{R}_{i+1} - \mathbf{R}_i\|_2$. Similar to the pair distances, side length observations are highly variant.

Pairs

The *Pairs* model uses hyperedges connecting two or three pairs of nuclei (four or six nuclei). The first four features measure pair-to-pair variation, while the latter two features use triplets of pairs to measure angles formed by the midpoints of the three pairs. The first pair-to-pair feature extends upon the use of pair distances to better describe the coiled worm. The ratio of sequential pair distances models the variation in width throughout the assigned nuclei (S1 Fig 2-A):

$$PDR_i = \frac{PD_i}{PD_{i+1}}. \tag{19}$$

Each feature's estimated mean is slightly greater than 1, indicating that, on average, the worm is widening from tail to head. Another easily interpreted distance is the length of the chords connecting sequential pair midpoints. This is a more robust measure of worm length as side lengths vary more based upon the worm's folding (S1 Fig 2-B):

$$MD_i = \|\mathbf{M}_{i+1} - \mathbf{M}_i\|_2. \tag{20}$$

The length of the chords connecting sequential pair midpoints is a more robust measure of worm length as side lengths vary more based upon the worm's folding. The cosine similarity is used to assess the degree to which sequential sides are pointing in the same direction (Fig 2-C):

$$\phi_i = \frac{(\mathbf{R}_{i+1} - \mathbf{R}_i) \cdot (\mathbf{L}_{i+1} - \mathbf{L}_i)}{\|\mathbf{R}_{i+1} - \mathbf{R}_i\|_2 \|\mathbf{L}_{i+1} - \mathbf{L}_i\|_2} \in [-1, 1]. \tag{21}$$

The final two pair-to-pair *Pairs* features attempt to model two different types of *twist* in the posture. The lateral and axial twists measures angles of rotation from lateral and posterior views, respectively (S1 Fig 2-D). Lateral twists can be expressed via ψ:

$$\mathbf{b}_1 = \frac{\mathbf{L}_{i+1} - \mathbf{L}_i}{\|\mathbf{L}_{i+1} - \mathbf{L}_i\|_2} \tag{22}$$

$$\mathbf{b}_2 = \frac{\mathbf{L}_i - \mathbf{R}_i}{\|\mathbf{L}_i - \mathbf{R}_i\|_2} \tag{23}$$

$$\mathbf{b}_3 = \frac{\mathbf{R}_i - \mathbf{R}_{i+1}}{\|\mathbf{R}_i - \mathbf{R}_{i+1}\|_2} \tag{24}$$

$$\mathbf{b}_4 = \frac{\mathbf{R}_{i+1} - \mathbf{L}_{i+1}}{\|\mathbf{R}_{i+1} - \mathbf{L}_{i+1}\|_2} \tag{25}$$

$$\mathbf{n}_1 = \mathbf{b}_1 \times \mathbf{b}_2 \tag{26}$$

$$\mathbf{n}_2 = \mathbf{b}_2 \times \mathbf{b}_3 \tag{27}$$

$$\mathbf{n}_3 = \mathbf{b}_3 \times \mathbf{b}_4 \tag{28}$$

$$\mathbf{c}_1 =< \mathbf{n}_1 \times \mathbf{n}_2, \mathbf{b}_2 > \tag{29}$$

$$\mathbf{c}_2 =< \mathbf{n}_1, \mathbf{n}_2 > \tag{30}$$

$$\psi_i = \frac{1}{\pi} atan2(< \mathbf{n}_1 \times \mathbf{n}_2, \mathbf{b}_2 >, < \mathbf{n}_1, \mathbf{n}_2 >)). \tag{31}$$

$$\tag{32}$$

Axial twists present between a sequence of two pairs calculates the angle obtained by projecting the chord linking pairs onto each other (S1 Fig 2-E):

$$\mathbf{b}_1 = \frac{\mathbf{L}_i - \mathbf{L}_{i+1}}{\|\mathbf{L}_i - \mathbf{L}_{i+1}\|_2} \tag{33}$$

$$\mathbf{b}_2 = \frac{\mathbf{R}_i - \mathbf{L}_i}{\|\mathbf{R}_i - \mathbf{L}_i\|_2} \tag{34}$$

$$\mathbf{b}_3 = \frac{\mathbf{R}_{i+1} - \mathbf{R}_i}{\|\mathbf{R}_{i+1} - \mathbf{R}_i\|_2} \tag{35}$$

$$\mathbf{b}_4 = \frac{\mathbf{L}_{i+1} - \mathbf{R}_{i+1}}{\|\mathbf{L}_{i+1} - \mathbf{R}_{i+1}\|_2} \tag{36}$$

$$\mathbf{n}_1 = \mathbf{b}_1 \times \mathbf{b}_2 \tag{37}$$

$$\mathbf{n}_2 = \mathbf{b}_2 \times \mathbf{b}_3 \tag{38}$$

$$\mathbf{n}_3 = \mathbf{b}_3 \times \mathbf{b}_4 \tag{39}$$

$$\mathbf{c}_1 =< \mathbf{n}_2 \times \mathbf{n}_3, \mathbf{b}_3 > \tag{40}$$

$$\mathbf{c}_2 =< \mathbf{n}_2, \mathbf{n}_3 > \tag{41}$$

$$\tau_i = \frac{1}{\pi} atan2(< \mathbf{n}_2 \times \mathbf{n}_3, \mathbf{b}_3 >, < \mathbf{n}_2, \mathbf{n}_3 >)). \tag{42}$$

$$\tag{43}$$

Angles along sides of the worm formed by triples of sequential nuclei approximate bend in the worm along each side. These angles are highly variant, in the same manner as side lengths in *Sides* (S1 Fig 1-B). Angles formed by pair midpoints exacerbate the computational burden as six nuclei are required, compare to three in a typical angle calculation, but the midpoint based angles are less variant than angles of each side (S1 Fig 2-F):

$$\Theta_i = \frac{180}{\pi} \arccos \frac{< \mathbf{M}_{i+1} - \mathbf{M}_i, \mathbf{M}_{i+2} - \mathbf{M}_{i+1} >}{\|\mathbf{M}_{i+1} - \mathbf{M}_i\|_2 \|\mathbf{M}_{i+2} - \mathbf{M}_{i+1}\|_2}. \tag{44}$$

Each angle $\Theta \in [0, 180]$ where 0 would denote the worm perfectly folded upon itself, and 180 would define a flat worm. A second set of angles aims to approximate the posterior to anterior bend in the worm. The angles ζ_i are defines as the angles formed by fitted planes intersecting between pair midpoints (S1 Fig 2-G):

$$\zeta_i = \frac{180}{\pi} \frac{< (\mathbf{R}_{i+1} - \mathbf{L}_{i+1}) \times (\mathbf{M}_{i+1} - \mathbf{M}_i), ((\mathbf{R}_{i+1} - \mathbf{L}_{i+1}) \times (\mathbf{M}_{i+1} - \mathbf{M}_{i+1})) >}{\|(\mathbf{R}_{i+1} - \mathbf{L}_{i+1}) \times (\mathbf{M}_{i+1} - \mathbf{M}_i)\|_2 \|(\mathbf{R}_{i+1} - \mathbf{L}_{i+1}) \times (\mathbf{M}_{i+1} - \mathbf{M}_{i+1})\|_2}. \tag{45}$$

Posture

The *Posture* model is comprised of all *Pairs* features as well as the features defined by the summations of each local feature measurement throughout the hypothesized posture. Full posture features give insight into the changes in the embryo's shape throughout late-stage embryogenesis (S1 Fig 3). Worm length follows an approximately logarithmic pattern.Total curvature follows a negative exponential pattern. Earlier on the worm is fatter and cannot bend as much. The worm elongates during development, allowing for sharper bends.
