# Supplementary figures and images for: An Exact Hypergraph Matching algorithm for posture identification in embryonic C. elegans
Source: PLoS One. 2022 Nov 29;17(11):e0277343. doi: 10.1371/journal.pone.0277343 (PMC9707761; doi:10.1371/journal.pone.0277343)

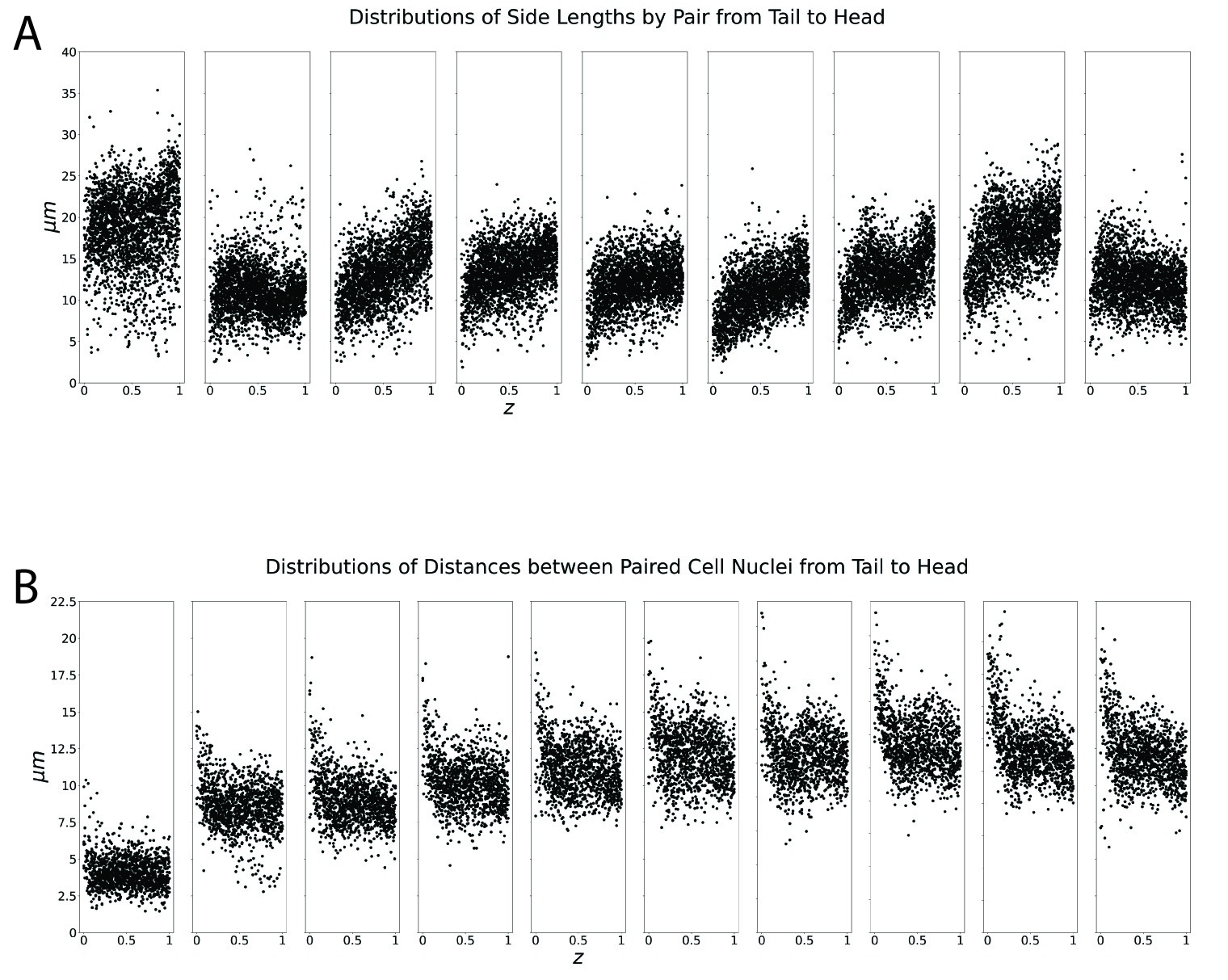

Supplement: S1 Fig — A) Distances between nuclei of lateral pairs. Notably, the tail pair distance (left-most panel) is constant throughout imaging. The tail pair distance informs the initial pair selection rule H1. B) Chord lengths along left and right sides of the posture. Both quadratic features show high variance. (TIF) [file pone.0277343.s002.tif]

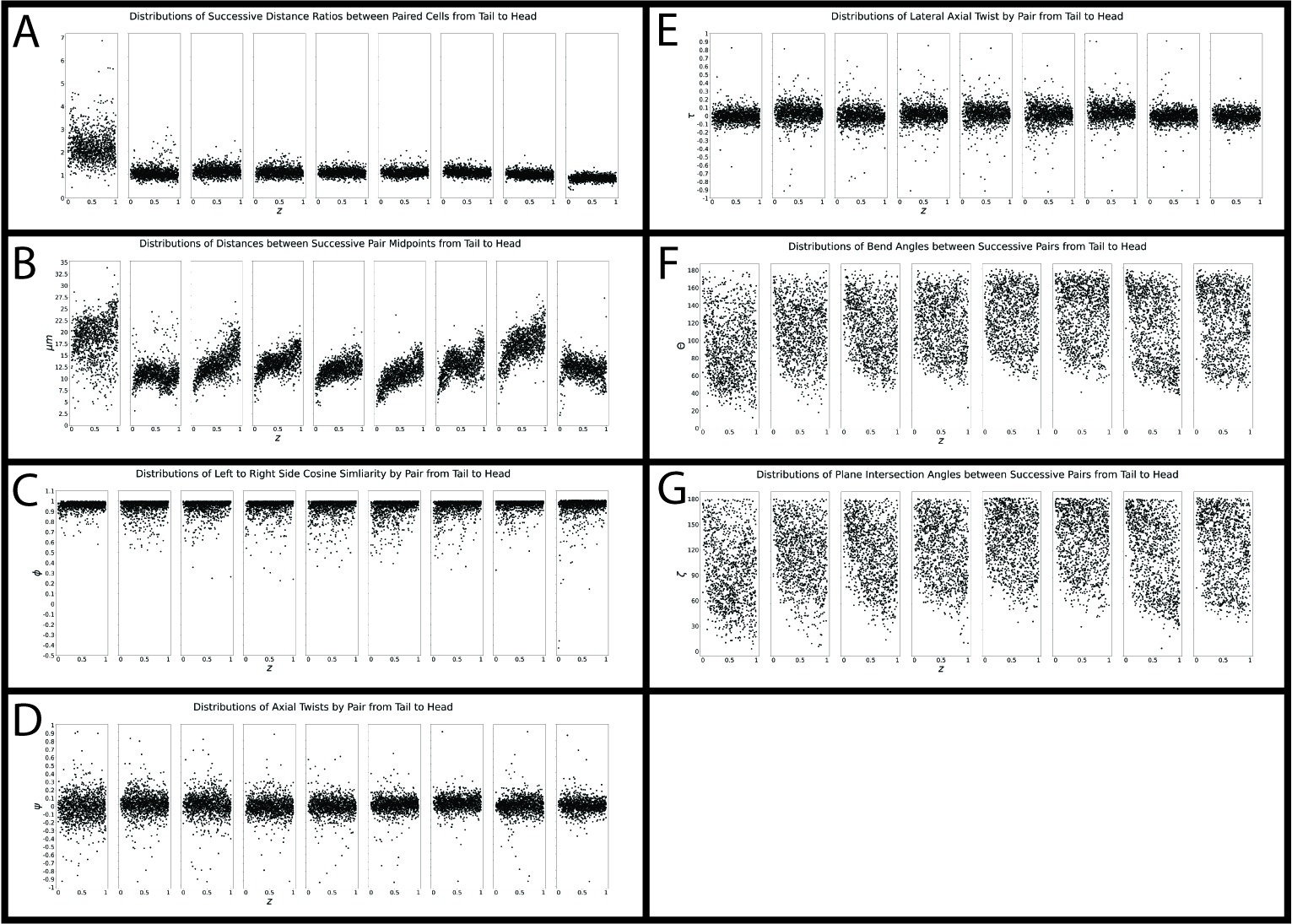

Supplement: S2 Fig — A) Ratios of pair distances. B) Distance between successive pair midpoints. C) Cosine similarities between successive left and right sides. D) Lateral axial twist angles. E) Axial twist angles. F) Midpoint bend angles. G) Planar intersection angles. (TIF) [file pone.0277343.s003.tif]

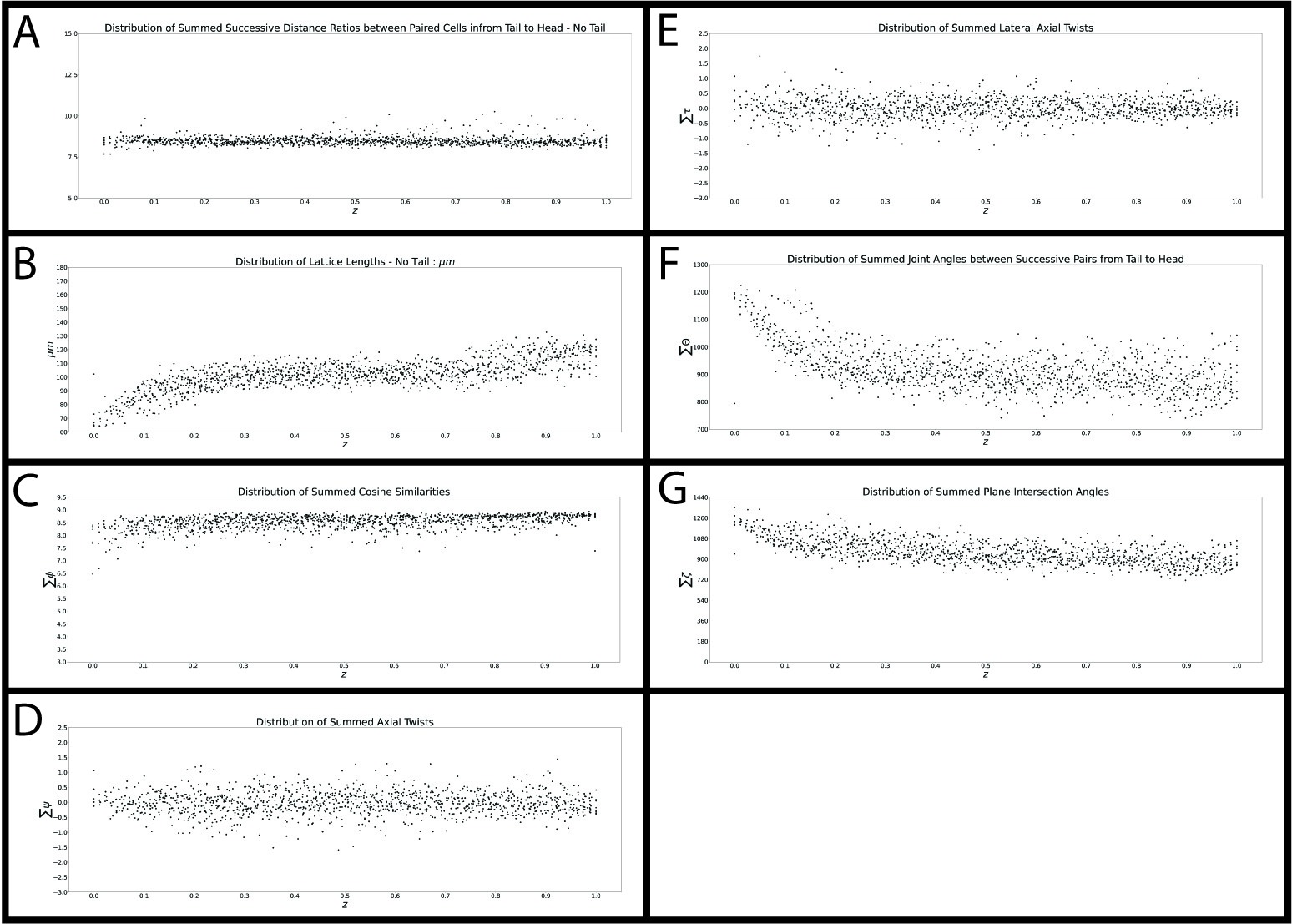

Supplement: S3 Fig — A) Summed ratios of pair distances. B) Summed distances between successive pair midpoints. C) Summed cosine similarities between successive left and right sides. D) Summed lateral axial twist angles. E) Summed axial twist angles. F) Summed midpoint bend angles. G) Summed planar intersection angles. (TIF) [file pone.0277343.s004.tif]
