# Supplementary material for: An Exact Hypergraph Matching algorithm for posture identification in embryonic C. elegans
Source: PLoS One. 2022 Nov 29;17(11):e0277343. doi: 10.1371/journal.pone.0277343 (PMC9707761; doi:10.1371/journal.pone.0277343)
Supplement: Attachment — Submitted filename: PLOS_EHGM_rebuttal_letter_resubmit.docx [file pone.0277343.s005.docx]

Laboratory of High Resolution Optical Imaging

13 South Drive Room G800

Bethesda Maryland, 20892

Dr. Akbar Ali

Academic Editor

PLOS ONE

Tuesday May 3, 2022

Dear Dr. Ali,

The co-authors and I thank you for handling our manuscript *An Exact Hypergraph Matching Algorithm for Posture Identification in Embryonic C. elegans*. In response to your feedback, we have addressed *PLOS* ONE’s style requirements and clarified the funding section inconsistencies, corrected the formatting, and addressed the reviewer’s comments. A point-by-point response follows. Line numbers are referenced in parentheses.

**Editor’s comments**

1. Please ensure that your manuscript meets PLOS ONE's style requirements, including those for file naming. The PLOS ONE style templates can be found at
   https://journals.plos.org/plosone/s/file?id=wjVg/PLOSOne_formatting_sample_main_body.pdf and 
   https://journals.plos.org/plosone/s/file?id=ba62/PLOSOne_formatting_sample_title_authors_affiliations.pdf.

We confirm that our submission has been formatted to the *PLOS* ONE publishing criteria. The manuscript was compiled using the *PLOS* Latex template. The figures are each uploaded according to the figure guidelines.

2. Tables Not In Manuscript File (in the case of file type PDF)’ send back text: 'Please include your tables as part of your main manuscript and remove the individual files. Please note that supplementary tables (should remain/ be uploaded) as separate "supporting information" files

The results tables, Tables 1, 2 & 3, are compiled in the main body of the manuscript. Our submission does not contain any supplementary tables.

3. We note that the grant information you provided in the ‘Funding Information’ and ‘Financial Disclosure’ sections do not match.

The Funding Information section has been updated in the revised submission. However, we were unable to edit the Financial Disclosure section. I wrote an amended Financial Disclosure section in an attached cover letter, but will reproduce the paragraph below:

“Andrew Lauziere's contribution to this research was supported in part by NSF award DGE-1632976. Radu Balan’s contribution to this research was supported in part by NSF under grants DMS-1816608 and DMS-2108900, and by Simons Foundation under Simons Fellows in Mathematics program. The funders had no role in study design, data collection and analysis, decision to publish, or preparation of the manuscript.”

**Reviewer’s comments**

**Major comments**

1: The definition of “posture identification” is a bit unclear, making it difficult to interpret the results. Although the “top x accuracy” and “cost ratio” are explained in the text, it would be easier for the layperson to understand if they were shown in schema.

We have edited the manuscript to clearly define posture identification in the Introduction (102), Results (187), and Methods (334-336) sections. We also designed a new figure (now numbered Fig. 3) to depict two postures in a 3D rendering GUI. In conjunction with previous figures (now numbered Fig. 1, Fig. 2, Fig. 4, and Fig. 6), readers will easily be able to follow the definition of posture as the identification of all seam cells. ­­­­­

The description of both the “top x accuracy” and cost ratio” metrics has been revised for clarity (173-186). The revised writing better define both terms and explains the intuition behind the cost ratio metric.

2: Table 1, 2 and 3 compare the performance of 4 methods (KerGM, QAP, Pairs and Pusture), but the reviewer (and maybe the majority of readers) cannot intuitively recognize which method is EHGM. Therefore, a supplementary explanation would be helpful.

This comment led us to reevaluate the way in which our results are presented. The Results section has been split into two subsections. The first section separates the search algorithm *EHGM* and the models we use (via *EHGM*) to identify posture. We hope the new organization, detail, and accompanying figure (Fig. 4) will help the readers recognize *EHGM* and ease them into the second Results section, a more traditional comparison of methods evaluated on the sample data.

3: The real microscopic image only appears in Figure 6. The reviewer believes that it would be very helpful to understand what each figure meant to say if the real pictures of nematodes were also displayed side-by-side in Figures 1 through 4.

We have addressed this concern with the addition of Fig. 3 in the Introduction section. The figure is described in a new subsection of the Introduction, *Overview of EHGM & Application to C. elegans*. This figure displays two rendered image volumes in which points are already shown in Fig. 1. The 2x2 grid shows the illuminated seam cell nuclei with and without manually annotated posture splines. Now Figs. 1, 3, and 4 all show data from the same image volumes, conveying the process of posture identification both manually and automatically via *EHGM*.

4: Figure 9, 10, 11 are no longer referenced in the manuscript. Therefore, it is unclear in what context and for what purpose these figures are presented in the study.

Figures 9, 10, and 11 depicted distributions of feature measurements used in *EHGM* models. These figures were only referenced in the manuscript’s supplement. The figures are now listed as supplementary figures (S1 Figs. 1, 2, 3) and removed from the main text of the manuscript accordingly.

**Minor comments**

1: Please indicate the full term of QAP (quadratic assignment problem?) when it first appears.

We now define the quadratic assignment problem (QAP) mathematically in the Introduction section (15-22). The acronym is presented in the definition.

2: In the legend of Fig. 10 and 11, some right parenthesis are missing.

These typos have been resolved.

3: While the research paper is of a very high level of content, it is also notable for its redundancy. The Results and Methods sections describe what should be stated in the Introduction and Discussion sections, which undermines readability. The reviewer recommends to write only results in the Results section and only methods in the Methods section.

In light of this comment, we have substantially revised the manuscript. They are mostly organizational in nature and aim to address both the redundancy and readability of the manuscript. We hope that each section reads more like that of a traditional research paper after moving material around, i.e., results in the Results section and methods in the Methods section, etc. In more detail:

The Introduction section now houses content moved from both the Results and Methods sections. The revised Introduction section better eases readers into the manuscript by describing the mathematical problem (2-47) and including a brief review of related research (48-79). The first subsection of Methods has been relocated and expanded upon to form the first part of the Introduction. A new subsection concludes the Introduction section, titled *Overview of EHGM & Application to C. elegans* (80-131). This subsection contains a summary of *EHGM* which was originally placed in the Results section. The subsection also introduces the necessary *C. elegans* biology for readers to follow the Results and Discussion sections.

The Results section is now split into two subsections, as noted in our response to major comment #2 above. Additionally, material describing the challenges to posture identification has been moved to the Discussion section (245-253).

We also heavily revised the Methods section. The section now contains two subsections describing *EHGM*, which are both unchanged from the reviewed manuscript. We have removed redundant information in the subsection *Posture Identification in Embryonic C. elegans*, leaving a couple paragraphs describing worm anatomy and imaging (326-347). We added detail concerning our application of *EHGM* and moved what was formerly Fig. 3 (now Fig. 9) to this Methods subsection to tie together the method and application.

The other authors and I find that the revised manuscript addresses both your comments and the reviewer’s critiques. We are grateful for all feedback as the manuscript is now in line with *PLOS* ONE publishing standards and readability has been improved. Thank you for your continued support of our manuscript.

Sincerely,

Andrew Lauziere (on behalf of all authors)

Department of Mathematics, University of Maryland College Park & NIBIB/NIH

[lauziere@umd.edu](mailto:lauziere@umd.edu)
