# Supplementary material for: An Exact Hypergraph Matching algorithm for posture identification in embryonic C. elegans
Source: PLoS One. 2022 Nov 29;17(11):e0277343. doi: 10.1371/journal.pone.0277343 (PMC9707761; doi:10.1371/journal.pone.0277343)
Supplement: Attachment — Submitted filename: rebuttal_letter.docx [file pone.0277343.s006.docx]

Laboratory of High Resolution Optical Imaging

13 South Drive Room G800

Bethesda Maryland, 20892

Dr. Miquel Vall-llosera Camps

Senior Editor

PLOS ONE

Tuesday September 27, 2022

Dear Dr. Vall-llosera Camps,

The co-authors and I thank you for assuming editorial responsibilities of our manuscript *An Exact Hypergraph Matching Algorithm for Posture Identification in Embryonic C. elegans*. We are aware of the journal’s efforts to procure both a suitable editor and reviewers. Further feedback from both reviewers has improved our manuscript. A point-by-point response follows.

**Additional Editor Comments:**

1. **PLOS ONE requires that submissions that present new methods as the primary focus of the manuscript must meet the criteria of utility, validation, and availability, which are described in detail at** [**https://journals.plos.org/plosone/s/submission-guidelines#loc-methods-software-databases-and-tools**](https://journals.plos.org/plosone/s/submission-guidelines#loc-methods-software-databases-and-tools)**.**

The manuscript primarily focuses on our novel algorithm, *EHGM*, and the inspiring application, posture identification in embryonic *C. elegans*. We find that our contributions achieve the three necessary criteria: utility, validation, and availability.

*EHGM* solves especially intricate optimization problems which are not often attempted due to their infeasibility. Particularly, we find that posture identification benefits from intricate models of seam cell relationships; *EHGM* was used to find the optimum under such models, returning improved posture accuracy over off-the-shelf methods. Utility was demonstrated on posture identification, with a preceding discussion on the nature of point-set matching problems which could also benefit from *EHGM* (lines 91-104).

Our method was empirically validated on posture identification, a cutting-edge problem in developmental biology. Posture identification was previously unapproachable given its complexity with respect to available methods. We compared two *EHGM* models (*Pairs* and *Posture*) to *KerGM*, a previously published graphical point-set matching algorithm. We found that *EHGM* with the *Posture* model approximately doubled the number of correct postures relative to *KerGM*, 56% to 27%. Further analysis showed that user seeding of the posterior-most cells improved accuracy to 72%. *EHGM* allows for arbitrarily intricate point-set matching models to be fit and applied, allowing for the development of more accurate posture identification models than *Pairs* or *Posture.*

We have made publicly available both the *EHGM* code and data used in our analyses on Github (<https://github.com/Lauziere/ehgm/>) free of charge. The algorithm and parameter fitting were written entirely in Python3 and use only NumPy and Pandas, a standard Python module for handling arrays and a module for handling data, respectively. The code is accompanied by a standard MIT License and meets other criteria necessary to meet the Open Source Definition.

In summary, *EHGM* was developed to approach an especially challenging point-set matching problem, which we hope inspires other researchers to revisit problems of similar complexity. We focus on posture identification, providing an open source implementation of *EHGM* and apply our method to all sample data discussed in the manuscript.

**Review Comments to the Author**

**Reviewer #1:**

1. **Does the title of Figure 1 correctly represent the content? It says that “low temporal resolution imaging necessitates posture identification”, but the reviewer thinks “high temporal resolution imaging rather necessitates posture identification”. “Failure of traditional tracking approaches” may better describe the content of the Figure 1.**

Here we intended to convey that the challenge in posture identification comes from the five-minute (‘low temporal resolution imaging’) gap between images. This five-minute gap is necessary to prevent phototoxicity at the high spatial resolution used in this study. Increasing the image capture rate could produce a posture identification problem more amenable to existing methods, as each seam cell would move very little relative to the size of the embryo, frame-to-frame. This would make posture identification more akin to a standard multiple object tracking problem. However, such an increase in the capture rate would exacerbate photobleaching and harm the embryo due to phototoxicity.

We agree Figure 1’s title did not convey our intent clearly. However, we do stand firmly that the low temporal resolution is what necessitates posture identification. The title has been updated to “Seam cells are used to recover *C. elegans* posture in high spatial resolution, low temporal resolution imaging” on line 126.

2. **Figure 3 is a visualization of Figures 1-B, so it is easier to understand when merged with Figure 1. Otherwise, it is recommended to rename it to Figure 2.**

We agree and have swapped the order of Figures 2 and 3. The text has also been rearranged to maintain cohesion; lines 127-136 now support Figure 2 while lines 137-148 support Figure 3.

3. **In all of Tables 1-3, it would be more formal and easier to understand if the rows and columns were interchanged. At first glance, it is difficult to understand what Top 1 to Top 10 represent, so a column of one higher level should be created to clearly indicate that they represent “Accuracy” (In the reviewer's personal opinion, either the Top 1 Accuracy or the Top 10 Accuracy should be sufficient to show in the Tables.).**

The original tables (previously Tables 1-3) have been transposed according to this suggestion; they are currently labelled Tables: 1 (line 227), 2 (line 238), and 4 (line 270). Table 3 (line 257) was introduced to exclusively show cost ratio and runtime information which was previously in Table 3. The cost ratio and runtime information pertaining to all samples together previously in Table 1 has been removed. The column label ‘Accuracy’ was also added above the first columns of Tables 1, 2, and 4 to better clarify the meanings of Top 1, Top 2, etc.

However, Top 1, 2, and 3 accuracies were retained to demonstrate how similar correct postures were to optimal postures across samples. Showing Top 1 and Top 10 alone would hide the decreasing nature of marginal accuracy improvements. The marginal changes from Top 3 onward were negligible, and so Top 5 and Top 10 were removed.

4. **It is not intuitively clear that the top chart and bottom chart of Table 2 correspond to the presence and absence of Q neuroblasts, respectively. The reviewer would like the authors to devise a better way to express this. Abbreviations for “Cost Ratio” and “Runtime” should be defined in legends for each table, or else they should be written in full terms.**

Table 2 now combines the two disjoint tables into one larger table. Each subtable has a spanning column to highlight pre-*Q* and post-*Q* samples (line 238). We found this to be approachable and more intuitive. We have also defined “Cost ratio” and “Runtime” in the legend to Table 3.

5. **The title of Table 3 says "seeding posterior pair identities reduce runtime", but does the Table really contain data on runtime?**

This typo has been corrected. The title of Table 4 (previously Table 3) has been updated to “Seeding posterior pair identities enabled more accurate posture identification.”

6. **Is the difference of accuracy between “Pairs” and “Posture” statistically significant?**

This comment prompted us to analyze the data more rigorously and correspondingly update our conclusions concerning *EHGM* model performances. Independent two sample t-tests were performed to compare mean accuracies between models across all samples and samples split by *Q* pair presence (lines 220-221 and 232-234). We found that while there was not a statistically significant difference between *Pairs* and *Posture* across all samples, *Posture* did outperform *Pairs* on pre-*Q* samples.

We then conducted two sample Kolmogorov-Smirnov tests to compare the runtime and cost ratio distributions (lines 246-248, 248-250, 250-253, 255-257). The results strengthened the previous result that *Posture* more effectively modeled posture identification than *Pairs* on pre-*Q* samples (line 246-248), but not on post-*Q* samples (line 248-250). We then argued that *Posture* outperformed the graphical model *Sides* (according to the cost ratio metric) on both pre-*Q* (lines 250-253) and post-*Q* samples (line 250-253). Our final test gave strong evidence that the distribution of *Posture* runtimes was higher than the distribution of *Pairs* runtimes on pre-*Q* samples, demonstrating a trade-off between performance and runtime (line 255-257).

Our additional analysis and updated tables better communicate the Results section of the paper. We thank Reviewer #1 for their valuable feedback.

Reviewer #2:

1. **The current version of the article is interesting and well-written. I only suggest to the authors putting convenient punctuation after each formula.**

We added proper punctuation on equations 1-6 (lines 14, 22, 37, 346, 354, and 367) in the manuscript as well as on equations in Supplement 1.

Additional changes:

Figure 5 has been redesigned to better follow-up Table 4. Most notably, the two plots have been merged into one. The similarity in accuracies and runtimes between the *Pairs* and *Posture* seeded simulations made for a distracting figure. We decided it would be better to compare accuracy and runtime between graphical models (represented by *Sides*) and hypergraphical models (represented by *Posture*). Now readers can intuitively compare the two models as a function of a priori information.

The other authors and I find that the revised manuscript addresses both your comments and the reviewer’s critiques. We are grateful for all feedback as the manuscript is now in line with *PLOS* ONE publishing standards and readability has been improved. Thank you for your continued support of our manuscript.

Sincerely,

Andrew Lauziere (on behalf of all authors)

Department of Mathematics, University of Maryland College Park & NIBIB/NIH

[lauziere@umd.edu](mailto:lauziere@umd.edu)
