# Supplementary material for: An Exact Hypergraph Matching algorithm for posture identification in embryonic C. elegans
Source: PLoS One. 2022 Nov 29;17(11):e0277343. doi: 10.1371/journal.pone.0277343 (PMC9707761; doi:10.1371/journal.pone.0277343)
Supplement: Attachment — Submitted filename: rebuttal_letter.docx [file pone.0277343.s007.docx]

Laboratory of High Resolution Optical Imaging

13 South Drive Room G800

Bethesda Maryland, 20892

Dr. Barry L. Bentley

Academic Editor

PLOS ONE

Monday October 24, 2022

Dear Dr. Bentley,

The co-authors and I thank you for assuming editorial responsibilities of our manuscript *An Exact Hypergraph Matching Algorithm for Posture Identification in Embryonic C. elegans*. We are aware of the journal’s efforts to procure both a suitable editor and reviewers. Further feedback from Reviewer #1 has produced the strongest version of our manuscript to date. A point-by-point response follows.

**Journal Requirements:**

**Please review your reference list to ensure that it is complete and correct. If you have cited papers that have been retracted, please include the rationale for doing so in the manuscript text, or remove these references and replace them with relevant current references. Any changes to the reference list should be mentioned in the rebuttal letter that accompanies your revised manuscript. If you need to cite a retracted article, indicate the article’s retracted status in the References list and also include a citation and full reference for the retraction notice.**

The authors and I confirm the reference list to be complete and correct.

**Review Comments to the Author: Reviewer #1**

**#1. Based on the update of the main manuscript, the reviewer thinks there is room to revise the ABSTRACT a little more. The current ABSTRACT begins with an explanation of point matching, but the reviewer thinks it would be easier for the reader to understand the flow of the discussion if the explanation began with the problems of posture estimation in C. elegans embryo. Also, how about adding some more concrete (or quantitative) experimental results?**

Both Dr. Bentley and Reviewer #1 agreed the abstract should be rewritten to lead with *C. elegans* and the posture identification problem. We have revised the Abstract accordingly. The Abstract now first introduces *C. elegans*, the twitching motions, and the how the seam cells are used to recover posture. Posture identification is then shown to be a crucial step to performing analyses in late-stage development. The problem is then viewed as a point-set matching problem, leading to our proposed method, *EHGM*. We conclude the abstract by citing experimental results demonstrating the efficacy of our approach.

**#2. To ensure the reproducibility of the experimental results, might the authors consider making the datasets and programs used in this study publicly available? Are there any plans to implement the proposed method as a plug-in or add-in function for image analysis software such as ImageJ ?**

The datasets and programs have been made publicly available since the manuscript’s initial submission via Github. The code is freely available and written in Python. We have announced the code’s availability in the Acknowledgements section (lines 453-454): “The code and data are freely available at <https://github.com/lauziere/EHGM>.” Here users can select one of the *EHGM* models: *Sides*, *Pairs*, or *Posture* and apply the method to all samples used in the study. We do not have plans to deploy the method other than what is provided on Github.

The other authors and I find that the revised abstract addresses Reviewer #1’s concerns. We are grateful for all feedback as the manuscript is now in line with *PLOS* ONE publishing standards and readability has been improved. Thank you for your continued support of our manuscript.

Sincerely,

Andrew Lauziere (on behalf of all authors)

Department of Mathematics, University of Maryland College Park & NIBIB/NIH

[lauziere@umd.edu](mailto:lauziere@umd.edu)
